# Effectiveness of Shacklock's Neural Mobilization for Acute and Sub‐Acute Lumbar Disc Prolapsed: A Randomized Controlled Trial

**DOI:** 10.1002/hsr2.70872

**Published:** 2025-05-26

**Authors:** Zahid Bin Sultan Nahid, Faruq Ahmed, Md Faruqul Islam, Zakia Rahman, Md Furatul Haque, Asma Arju, Md Rafiqul Islam, Golam Moula, Bibekanonda Sarker

**Affiliations:** ^1^ Department of Physiotherapy SAIC College of Medical Science and Technology Dhaka Bangladesh; ^2^ Department of Physiotherapy Centre for the Rehabilitation of the Paralysed (CRP) Dhaka Bangladesh; ^3^ Department of Physiotherapy Dhaka College of Physiotherapy Dhaka Bangladesh; ^4^ Department of Physiotherapy State College of Health Sciences Dhaka Bangladesh; ^5^ Directorate General of Health Sciences (DGHS) Dhaka Bangladesh

**Keywords:** low back pain, lumbar disc prolapse, physical therapy, Shacklock's neural mobilization

## Abstract

**Background and Aims:**

Lumbar disc prolapse (LDP) is a more severe health issue worldwide. Shacklock's neural mobilization is a useful technique for treating prolapsed discs. The study aimed to examine the effectiveness of Shacklock's neural mobilization for acute and subacute LDP.

**Methods:**

The study was a double‐blinded, randomized controlled trial. A total of 42 individuals with LDP were allocated randomly by computer‐generated numbers to an experimental and a control group. The experimental group got neural mobilization along with usual physiotherapy treatment. The control group got just usual physiotherapy. Postural education was given for both groups in sitting and standing positions. The total number of therapy sessions was eight, with a frequency of four sessions per week for a duration of 2 weeks. The Dallas pain questionnaire (DPQ) and Oswestry disability index (ODI) questionnaire were measured on the initial day and after eight sessions of treatment. The data was analyzed through paired *t*‐test, unrelated *t*‐test, chi‐square, and Mann‐Whitney U test.

**Results:**

A significant improvement of pain in different positions and disability was demonstrated in the within‐group analysis by paired *t*‐test, whereas no significant improvement was found in the between‐group analysis by independent sample *t*‐test. The significant change was found on the DPQ score except for two variables. There was no statistically significant association between disability status and gender (*p* = 0.69) and between disability status and BMI (*p* = 0.41). A significant difference (*p* = 0.000) was found in both scales.

**Conclusion:**

Shacklock's neural mobilization, along with standard physiotherapy techniques, had a significant impact on pain and disability for people with acute and subacute LDP.

**Trial Registration:** Zahid Bin Sultan Nahid: CTRI/2023/04/051283

## Summary

- Shacklock's neural mobilization is a useful technique for treating prolapsed discs.
- The significant changes found in pain intensity between pretest and posttest in the experimental group.
- Shacklock's neural mobilization had a significant impact on pain and disability for people with acute and subacute prolapsed disc patients.

## 1 Introduction

Low back pain (LBP) is the most prevalent health problem, which is responsible for increased healthcare expenses due to job absences and incapacity. LBP ranks fifth among illness categories in terms of hospital care expenditures [1]. This condition may arise from a prolapsed disc, hypertrophic facet or surrounding ligaments, spondylolisthesis, or, exceptionally, malignancy or infection [2]. The lumber disc prolapse (LDP) is the primary source of both back pain and radiculopathy [3]. About 90% of the time, lumbar radiculopathy is caused by an LDP that compresses nerve roots [4].

The LDP, a prevalent musculoskeletal condition, affects almost 10% of the global population [5]. The incidence of LDP in the lumbar spine is highly variable, primarily due to the characteristics of the population under investigation. Annual rates vary from 2.2% in the general population to as high as 34% in certain occupational categories [6]. Men have a larger likelihood of experiencing LDP during their fourth decade of life, whereas women had higher rates throughout their fifth and sixth decades of life [4].

The prevalence of LDP is the leading cause of functional impairment on a global scale, affecting around 1%–3% of the population [7]. Lumbar prolapsed disc is responsible for 60%–80% of the total number of cases of low back pain that occur over a person's lifetime in the general population [8]. The incidence of low back pain each year is said to be between 4% and 93%. In India, 23.19% of the population suffers from lumbar radiculopathy [9, 10]. Additionally, due to axial stress during movement, almost 85% of those with LBP also had further protrusion at the same level [11]. LBP leads to psychological alterations and morbidity, including disability, reduced engagement in both social and physical activities, and the development of excessive fear‐avoidance behavior [12]. The amount of disability caused by low back pain is increasing, resulting in a significant economic burden that surpasses $50 billion a year in the United States alone [13].

Neural mobilization is a hands‐on method aimed at reinstating the adaptability of the nervous system, which refers to the capacity of structures surrounding the nerves to move in relation to other similar structures [14]. According to Michael Shacklock's 2005 description, neurodynamic mobilization promotes the restoration of nerve cells' normal physiological function, reduces pain, and enhances performance while also helping the nervous tissue itself regain its capacity to withstand stress and strain [15]. Numerous studies have shown that neural mobilization is a useful technique for treating prolapsed discs or low back pain [16].

Due to the existing ambiguity about the most efficient physiotherapy method, further study is required to investigate the combination of different treatments in individuals with a distinct clinical profile. There are several therapeutic options for managing individuals with LBP; however, neural mobilization is a recently established strategy. This study investigates the effectiveness of neural mobilization during the first phase due to the lack of data in this period. Although there has been a lot of study on the usefulness of neural mobilization in patients with LBP, the specific therapy dose, including the level of intensity, duration, and number of repetitions, has not been fully determined. The aim of this study was to examine the effects of an eight‐session Shacklock's neural mobilization on pain intensity and disability status. The research also sought to examine the association between pain and disability with BMI and gender.

## 2 Methodology

### 2.1 Study Design

This is a double‐blinded, randomized controlled trial (RCT) design study where the assessor, physiotherapist, and participants were blinded. The researcher has conducted the study with an experimental and a control group with an aim to compare the between two groups.

### 2.2 Ethical Considerations

The ethical requirements of the World Health Organization (WHO), Bangladesh Medical Research Council (BMRC), and Centre for the Rehabilitation of the Paralysed (CRP) were followed. Before participating, patients got complete information about the research aims and protocol and provided signed informed consent. Administrative entities of the CRP ethics committee and the Institutional Review Board (IRB) authorized the project. The registration number is CRP/BHPI/IRB/10/2024/668. In addition, the trial was registered by the Clinical Trials Registry‐ India (CTRI/2023/04/051283).

### 2.3 Study Setting and Participants

Individuals with LBP and radiculopathy diagnosed as herniated discs by MRI at the musculoskeletal unit of the physiotherapy department at CRP between November 2022 and April 2023 were invited to take part in this study. Because these patients came to CRP from all over Bangladesh, from all economic groups for comprehensive rehabilitation, it reflects the entire population. Patient eligibility criteria were patients with acute and subacute single or multiple levels of lumbar disc prolapse evident in MRI [17, 18], both genders, with ages between 25 and 55 years [19], and patients with prolapseddiscs with symptoms of reduced closing dysfunction [16]. Subjects were excluded if they had any history of surgery for lumbar disc prolapse, suffered from serious pathological disease, for example, tuberculosis, tumour, or infection, had a history of fracture to the spine, were pregnant, were medically unstable patients, or had any condition where physiotherapy is contraindicated.

### 2.4 Randomization

**Figure 1:**
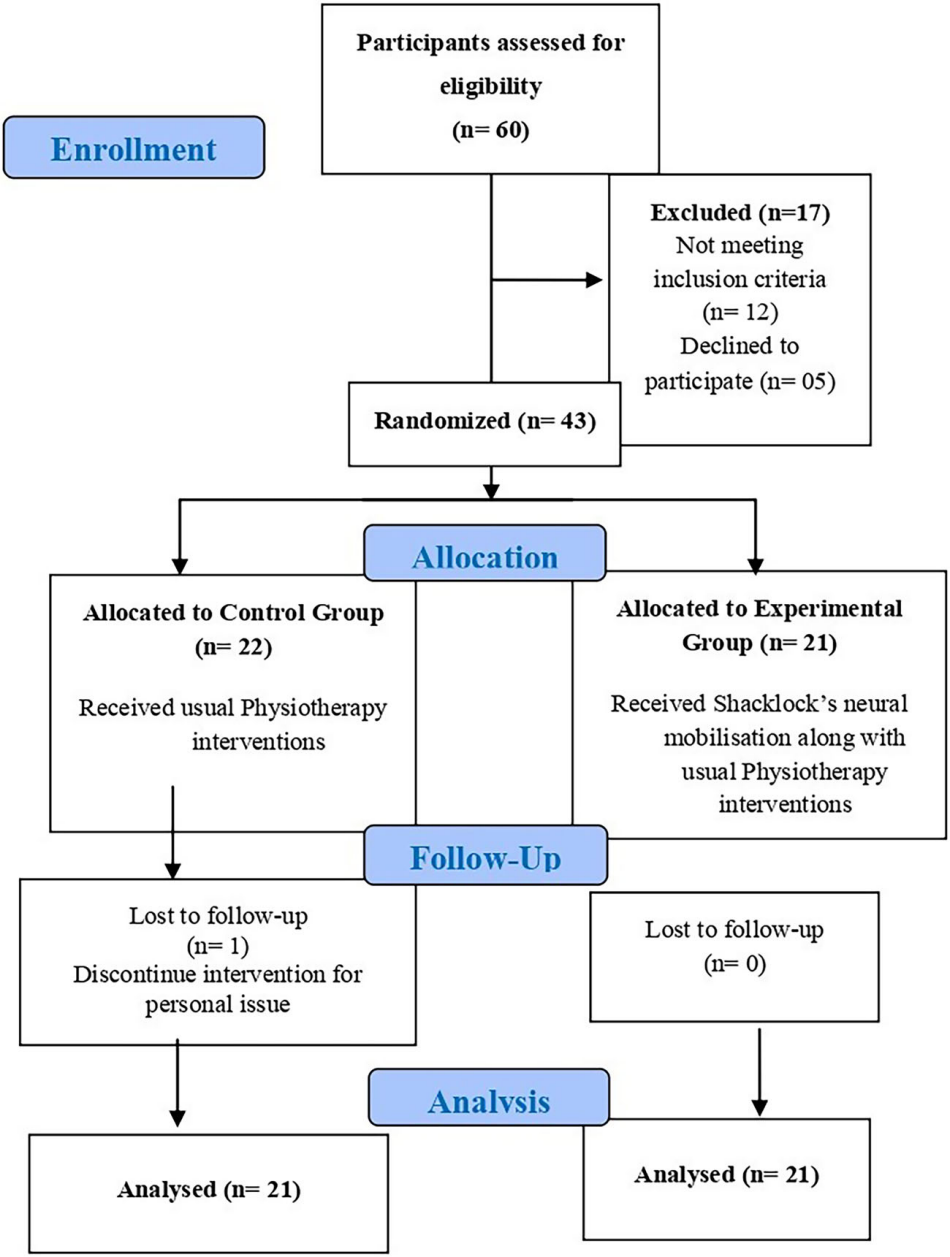
Depicts the research design and group distribution.

Random assignments enrolled 43 participants in either the experimental or control groups. Patients who satisfied the inclusion criteria were randomly recruited from the outpatient musculoskeletal unit of the physiotherapy department. Double blinding was used in this investigation. After sampling, the researcher randomly allocated individuals by computer‐generated random allocation to trial and control groups to increase the thesis's internal validity. The study was reported based on Consolidated Standards of Reporting Trials (CONSORT) statements for presenting parallel group randomized trials and interventions of neural mobilization. Figure 1 depicts the research design and group distribution.

### 2.5 Measurement

The researcher collected data through semi‐structured questionnaires and face‐to‐face interviews with different types of data collection tools. The researcher has used the Dallas pain questionnaire (DPQ) by using a visual analogue scale (VAS) for pain measurement in different working positions and also activities; the Oswestry disability index (ODI) questionnaire was used for disability measurement, and the structural questionnaire was used for socio‐demographic indicators. The interview was conducted before and after eight sessions of treatment. The researcher was to determine 42 participants understanding of the questions by observing their facial expressions.

### 2.6 Interventions

The experimental group got neural mobilization along with usual physiotherapy treatment, including McKenzie concept directional treatment procedures according to patients' conditions and basic physiotherapy treatment like pelvic floor, back and leg muscle strengthening, postural advice, electrotherapy modalities, and also given home advice. In the control group, participants were given only usual physiotherapy treatment. Postural advice was given in sitting and standing to both group participants. Bothgroups received therapy 4 days a week for 2 weeks. Treatment has been given by four 5‐year‐experienced qualified physiotherapists, and among them, two were trained in neural mobilization. The researchers arranged special training on neural mobilization and usual care.

#### 2.6.1 Treatment Protocol

The neural mobilization treatment protocol was developed by Michael Shacklock, and usual physiotherapy intervention was provided by CRP. Table 1 shows the following treatment protocol.

**Table 1:** Shacklock's neural mobilization and usual physiotherapy treatment protocol.

Experimental group (SNM along with UPT)	Control group (UPT intervention)
**Shacklock's neural mobilization with usual physiotherapy:** Progression (P)‐1: Static opener P‐2: Dynamic opener P‐3: Closing mobilization P‐4: Sliding P‐5: Tensioner P‐6: Closing with tensioner **Postural education and home exercises.**	**McKenzie approach**: 1 set of 10 repetitions performed in every 2 h (directional preference). **Lumbar spinal mobilization:** 30–60 oscillation per minute in every segment performed in each session. **Soft tissue release technique:** Performed 10 min in each session. **Lower back, pelvic floor and core muscles stabilization exercises:** 8–12 repetition of 1 set with 10 s hold twice daily. **Postural education and home exercises.**

Abbreviations: SNM, Shacklock's neural mobilization; UPT, usual physiotherapy.

### 2.7 Data Analysis

The statistical analysis was conducted using IBM's statistical program for social science (SPSS) version 22. A Mann‐Whitney U test was used to compare the baseline variability among the categorical data. Used paired *t*‐test to measure within‐group mean difference and Wilcoxon signed rank test to calculate between‐group mean differences. Spearman's correlation test was used to show the association among different variables. Two‐sided 95% confidence intervals were generated for the study. A significance level of *p*‐value < 0.05 was applied.

## 3 Results

The randomized controlled trial finally enrolled 40 patients as a sample. All patients finished the trial. The initial clinical characteristics of the study subjects are presented in Table 2. Each group's baseline characteristics were comparable. Since there were no adverse consequences, everyone accepted the intervention nicely. The mean age ± SD of the experimental and control groups were 41.48 ± 8.86 and 39.41 ± 10.31, respectively. The experimental group's mean BMI ± SD was 25.03 ± 2.91, while the control group's was 25.19 ± 4.15. The experimental and control groups' pain intensity ± SD, as measured by VAS, were 6.58 ± 1.56 and 6.67 ± 1.38, respectively. The experimental group's disability status ± SD was 54.42 ± 14.80, while the control group's was 52.63 ± 13.75.

**Table 2:** Baseline characteristics of the participants.

Variables	Experimental group *n* (=21)	Control group (*n* = 22)	*p* value
**Age (years) ± SD**	41.48 ± 8.86	39.41 ± 10.31	0.33
**Gender, *n* (%) (Male; Female)**	13 (61.9%); 08(38.1%)	14 (63.6%); 08 (36.4%)	0.91
**Height (m) Mean ± SD**	1.64 ± 0.06	1.63 ± 0.06	0.79
**Weight (kg) Mean ± SD**	65.90 ± 10.35	67.36 ± 11.72	0.94
**BMI (kg/m^2^) Mean ± SD**	25.03 ± 2.91	25.19 ± 4.15	0.42
**Duration of suffering, *n* (%)**			
Acute (> 4 weeks)	03 (14.3%)	06 (27.3%)	0.31
Sub‐acute (4–12 weeks)	18 (85.7%)	16 (72.7%)	
**Pain intensity ± SD** (Mean VAS in 10 cm)	6.58 ± 1.56	6.67 ± 1.38	0.35
**Disability status ± SD** (Mean ODI % ± SD)	54.42 ± 14.80	52.63 ± 13.75	0.58

### 3.1 Pain Intensity Between Group Comparisons

A Wilcoxon signed rank test has been used to measure the differences of the pre‐test Dallas pain questionnaire (10 cm VAS) between control and experimental groups, and most of the variables were significant differences found on the Posttest Dallas pain score between the two groups except back stiffness (where *Z* = −1.519, which is based on positive ranks and *p* = 0.129) and changes office for back pain (where *Z* = −2.326, which is based on positive ranks and *p* = 0.062) (Table 3).

**Table 3:** Dallas questionnaire (pre and post assessment by Wilcoxon signed‐rank test).

Serial	Variables	Median (IQR)		*p* value
		Pre test	Post test	
Pair 1	Pain intensity	6.6	3.3	0.000***
Pair 2	Pain intensity at night	5.6	2.6	0.000***
Pair 3	Pain intensity with lifestyle	6.6	3.2	0.000***
Pair 4	Pain at twisting	5.3	3.5	0.010**
Pair 5	Back stiffness	4.4	3.4	0.129
Pair 6	Pain severity after walking	6.3	3.3	0.000***
Pair 7	Pain keeps from standing still	5.3	2.7	0.000***
Pair 8	Pain during walking	6.0	3.3	0.000***
Pair 9	Pain severity at forward bending	6.3	3.4	0.000***
Pair 10	Pain allows sit in upright hard chair	5.3	2.8	0.000***
Pair 11	Pain allows sit in soft arm chair	5.2	2.5	0.000***
Pair 12	Pain lying in bed	4.8	2.1	0.000***
Pair 13	Pain limit normal life style	6.5	3.2	0.000***
Pair 14	Pain interferes with work	6.5	3.2	0.000***
Pair 15	Changes office for back pain	5.3	3.8	0.062

*Note:* Here, the Level of significance is (< 0.05), * = 0.05, ** = 0.01, and *** = 0.000.

**Figure 2:**
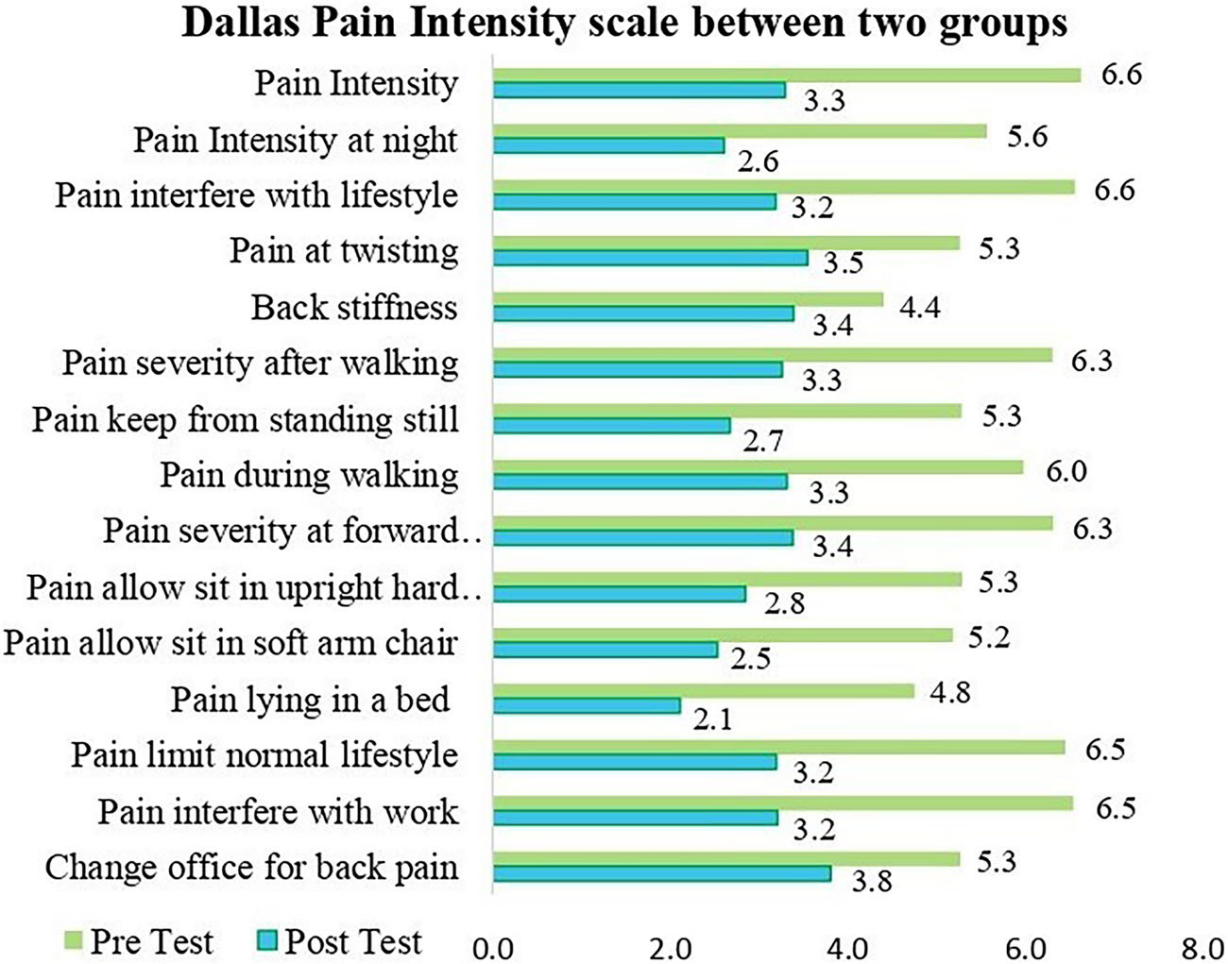
Posttest mean pain between both groups.

Figure 2 illustrates the mean value of the pre‐test and Posttest of the Dallas pain intensity scale between both groups. In this regard, the null hypothesis was rejected and the alternative hypothesis accepted. It has been explored that there was significant change found on the Dallas pain score, except for two variables (back stiffness and change in workplace variable).

### 3.2 ODI Score (Percentage) Between Two Groups

**Figure 3:**
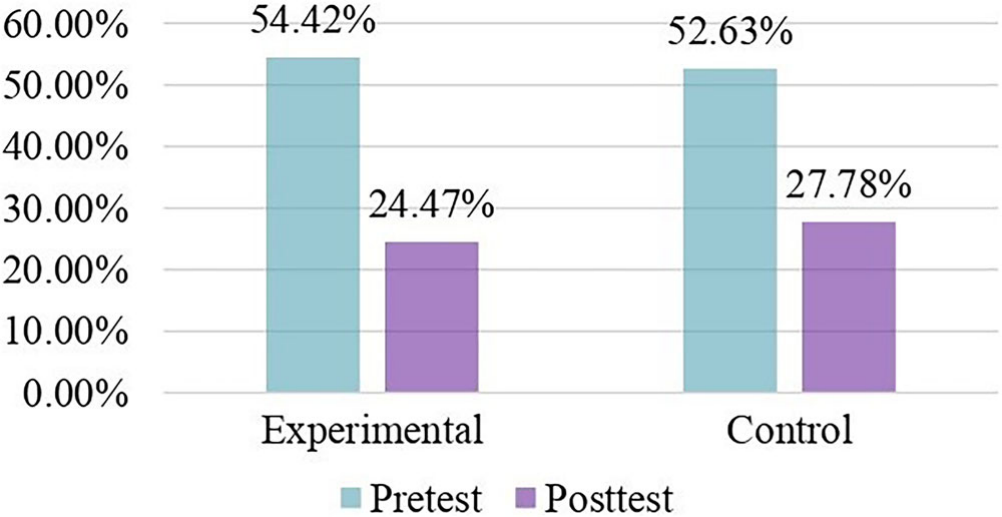
Mean ODI score (percentage) between two groups.

In Figure 3, the experimental group, pretest and posttest mean ODI score was 54.42% and 24.47%. Contrarily, in the control group, pretest and posttest mean ODI scores were 52.63% and 27.78%, respectively.

### 3.3 Compare Between Both Groups on Pain and Disability Score After Shacklock's Neural Mobilization

Before and immediately following the neural mobilization, the median pain and disability score were compared using a Wilcoxon signed‐rank test. A significant difference was found in the results (*p* = 0.000) in both scales, whereas the median pain and disability score after neural mobilization (median = 3.3; 19) was lower than the median pain and disability score before Shacklock's neural mobilization (median = 6.5; 52) (Table 4).

**Table 4:** Wilcoxon signed‐rank test between both groups on pain and disability score.

Variables	Median (IQR)		*p* value
	Pre test	Post test	
Dallas pain scale	6.5	3.3	0.000***
ODI (LBP)	52	19	0.000***

*Note:* Here, the Level of significance is (< 0.05), * = 0.05, ** = 0.01, and *** = 0.000.

### 3.4 Association Between Patient's Rated General Pain (cm) and ODI With Gender and BMI by Spearman's Correlation

Table 5 showed that there was no statistically significant association between patient‐rated general pretest pain (cm) and gender (*p* = 0.37), or between patient‐rated general pain (cm) and BMI (*p* = 0.41). As well, there was no statistically significant association between disability status and gender (*p* = 0.69) or disability status and BMI (*p* = 0.41).

**Table 5:** Cross tabulation between patients rated general pain and ODI with gender and BMI.

Variable 1	Variable 2	*p* value	Interpretation
General pain intensity (10 cm VAS)	Gender	0.37	No significant association
	BMI	0.41	No significant association
ODI	Gender	0.69	No significant association
	BMI	0.41	No significant association

*Note:* Here, the Level of significance is (< 0.05), * = 0.05, ** = 0.01, and *** = 0.000.

Table 6 showed that means the null hypothesis has been rejected and the alternative hypothesis accepted in both groups. It has been explored that there is a significant change found in the ODI score in both the experimental and control groups. According to the statistical test revealing no significant difference found between the pre‐test of the experimental and control groups in the ODI score. Shacklock's neural mobilization has no significant (< 0.05) impact on disability status.

**Table 6:** Disability status within and between group comparisons.

Disability status (Pre and post assessment‐paired‐*t* test)								
				Experimental group			Control group	
**Serial no.**	**Variables**			***t***	***p* value**	**df**	***t***	***p* value**
Pair 1	ODI			6.053	0.001***	20	4.820	0.001***

**Disability status (pre and post assessment Unpaired‐*t* test**)								
		**Pre assessment**				**Post assessment**		
**Variables**		***t***	***p* value**		**df**	***t***	***p* value**	**df**
ODI		0.362	0.72		41	−0.565	0.57	40

*Note:* Here, the Level of significance is (<0.05), * = 0.05, and ** = 0.01, *** = 0.001.

The unpaired “*t*” test has been used to measure the differences of the posttest DPQ (10 cm VAS) between the control and experimental groups, and there were no significant differences found in the posttest Dallas pain score between the two groups. This can be uttered that, between groups analysis found no significant difference on pain.

The paired sample “*t*” test has been determined to measure the changes in pain intensity between the pretest and posttest in the experimental group. It has been explored that there were significant changes found on the Dallas pain score except for two variables of pain: keeping away from twisting and a change in the workplace variable.

## 4 Discussion

The study's results demonstrate that Shacklock's neural mobilization exhibited substantial enhancements in both pain reduction and functional impairment compared to neural mobilization with conventional treatment (experimental group) and conventional therapy alone (control group). This supports the idea that neural mobilization treatments have a substantial impact on the treatment of lumbar radiculopathy. Sharma conducted a study comparing the effects of neurodynamic methods with traditional physiotherapy on athletes with lumbar radiculopathy. The study found that neurodynamic techniques were more helpful in lowering pain and functional handicap [20].

Neural mobilization strategies are crucial in the treatment of acute and subacute LBP caused by herniated disc discomfort. There is abundant evidence to substantiate the idea that neurodynamic testing causes the nerve bed to stretch, and this stretching is linked to nerve gliding. Stretching the nerve bed may cause the nerve to elongate, leading to heightened tension and intraneural pressure [21]. Neural mobilization is based on the idea that changes in the mechanics or physiology of the neurological system may cause dysfunctions in other systems or in the musculoskeletal tissues that are connected to it. Our approach involves using the neural mobilization method to alleviate pain and increase flexibility in the nervous system. The goal is to enhance neurodynamics and restore the flow of axoplasm, ultimately restoring the balance of nerve tissue and reducing pain. This treatment also facilitates the regaining of joint mobility [22].

A trial was conducted with neural mobilization along with motor control exercises for the experimental group by an experienced physiotherapist twice weekly for 8 weeks. The control group received only motor control exercises. The study concluded that neurodynamic mobilization in a motor control exercise program leads to reductions in neuropathic symptoms in subjects with lumbar disc prolapse [23]. One systematic review was carried out to evaluate the effectiveness of neural mobilization in pain, disability, and function in adults with low back pain, where six of the eight studies found positive effects on pain, disability, and function. It is revealed that neural mobilization is an effective tool for short‐term improvements in pain, function, and disability associated with low back pain [24].

Both of the groups exhibited a significant reduction in the ODI scale score with clinical importance. Both groups of subjects engaged in lumbar stabilization exercises, resulting in enhancements in functional impairment. The research demonstrated that lumbar stabilization exercise is superior to conservative therapy in enhancing functional impairment [25]. Nevertheless, several studies provide evidence that combining neural mobilization with lumbar stability exercises is more efficacious in improving functional impairment compared to exercises for lumbar stability alone [26, 27, 28]. Few of the studies supported that approximately eight to twelve therapeutic sessions for two to 4 weeks of interventions are ideal for neural mobilization and relative outcomes [27, 28]. The current investigation observed no difference in ODI decrease between the two groups. It is concluded that neural mobilization is an effective tool for short‐term improvements in pain, function, and disability associated with low back pain.

## 5 Limitation

The study was conducted with a limited number of subjects and a lack of long‐term follow‐up. Data was collected from only two clinical settings, which is in opposition to generalizability and might influence the findings. Another limitation is that about 25% of participants had some kind of comorbidity, likely diabetes mellitus, hypertension, or both, which might influence the outcome. There is a lack of consensus in the literature regarding the frequency and number of repetitions of neural mobilization techniques. In future studies, we recommend assessing the joint range and psychological state of the participants. Similar studies with a large sample size and a follow‐up session need to be involved.

## 6 Conclusion

The study found that Shacklock's neural mobilization, along with standard physiotherapy techniques, had a big impact on pain and disability after eight sessions of treatment for people with acute and sub‐acute LDP. A significant difference was found in pain and disability status. It is revealed that neural mobilization found improvements in pain, physical function, and disability with prolapsed discs.

## Author Contributions

**Zahid Bin Sultan Nahid:** conceptualization, methodology, writing – original draft. **Faruq Ahmed:** methodology, writing – original draft. **Md Faruqul Islam:** validation, visualization, supervision. **Zakia Rahman:** conceptualization, writing – review and editing. **Md Furatul Haque:** funding acquisition, investigation, resources. **Asma Arju:** software, project administration. **Md Rafiqul Islam:** investigation, writing – review and editing, writing – original draft. **Golam Moula:** formal analysis, project administration. **Bibekanonda Sarker:** data curation, validation.

## Ethics Statement

This study was approved by the Institutional Review Board and the Ethics Committee of Bangladesh Health Professions Institute (BHPI), Dhaka, Bangladesh (CRP/BHPI/IRB/10/2024/668).

## Conflicts of Interest

The authors declare no conflicts of interest.

### Transparency Statement

The lead author Zahid Bin Sultan Nahid affirms that this manuscript is an honest, accurate, and transparent account of the study being reported.

## Data Availability

The data that support the findings of this study are available from the corresponding author upon reasonable request.
